# Cross-Cultural Comparison of ICD-11 Adjustment Disorder Prevalence and Its Risk Factors in Japanese and Lithuanian Adolescents

**DOI:** 10.3390/brainsci12091172

**Published:** 2022-08-31

**Authors:** Kazuaki Abe, Ieva Daniunaite, Inga Truskauskaitė-Kunevičienė, Kazumi Sugimura, Paulina Zelviene, Shogo Hihara, Yuka Kamite, Evaldas Kazlauskas

**Affiliations:** 1 Graduate School of Humanities and Social Sciences, Hiroshima University, Kagamiyama, Hiroshima 739-8524, Japan; 2 Center for Psychotraumatology, Institute of Psychology, Vilnius University, 01300 Vilnius, Lithuania

**Keywords:** adjustment disorder, adolescents, cross-cultural, socio-interpersonal model

## Abstract

Background: Although there is increasing knowledge about adjustment disorder (AjD) based on the new diagnostic criteria of the International Classification of Diseases (ICD-11), less is known on AjD in adolescents. This study aimed to examine the prevalence of AjD and its risk factors in Japanese and Lithuanian adolescents. Methods: The cross-sectional study sample comprised 1745 adolescents from Japan (*n* = 913) and Lithuania (*n* = 832). AjD was assessed using the Adjustment Disorder New Module-8 (ADNM-8). We compared the prevalence of AjD in Japanese and Lithuanian adolescents. Using multinominal logistic regression analysis, we examined the effects of age, gender, socioeconomic status, and cumulative stressors as societal and cultural factors, resilience as an intrapersonal factor, and loneliness and perceived support as interpersonal factors on adolescent AjD. Results: The prevalence of probable AjD was 11.7% in Lithuanian adolescents and 6.9% in Japanese adolescents. Gender, socioeconomic status, cumulative stressors, resilience, loneliness, and perceived positive social support were each significantly associated with AjD risk. Conclusions: This cross-cultural comparative study revealed characteristics of the stressors and prevalence of AjD among Japanese and Lithuanian adolescents. In terms of the socio-interpersonal framework model for the stress–response syndrome, sociocultural, intrapersonal, and interpersonal factors were found to be risk factors associated with AjD in adolescents.

## 1. Introduction

Adjustment disorder (AjD) is a maladaptive reaction to an identifiable psychosocial stressor or multiple stressors (e.g., divorce, illness or disability, socio-economic problems, conflicts at home or work) that usually emerge within a month of the exposure to the stressor [1]. The diagnostic criteria for AjD were updated with the revision of the 11th edition of the International Classification of Diseases (ICD-11), which proposed two core symptoms of AjD: (1) preoccupation with a stressor; and (2) failure to adapt. These new proposals have led to an emphasis on the development of new assessment tools for AjD [2].

The Adjustment Disorder New Module 20 (ADNM-20; [3]) is a specific AjD diagnostic measure developed based on Maercker et al.’s theory-driven diagnostic concept [4]. The ADNM measures the two core symptoms of AjD proposed by ICD-11. Several studies have demonstrated the internal consistency, test–retest reliability, and discriminant and concurrent validity of the ADNM-20 [3,5]. There is also an 8-item brief version (ADNM-8) and a 4-item ultra-brief version (ADNM-4), both of which have demonstrated their diagnostic validity [6]. 

The prevalence of AjD in a sample of various cultures and communities has been reported using ADNM based on ICD-11 diagnostic definitions. For example, the prevalence of AjD was found to be 0.9–2.0% in a representative sample of the German general population [7,8] and 2.3% among the elderly in Switzerland [9]. In a sample of the Lithuanian general population exposed to life stressors (*n* = 649), the prevalence of AjD was 16.5% [10]. Among a population-representative sample of Chinese citizens, amid the civil unrest in Hong Kong in July 2019, the prevalence of probable AjD was 20.5% [11]. However, the majority of previous studies of ICD-11 AjD were conducted in adult populations, pointing to a lack of knowledge about AjD and its risk factors among child and adolescent populations [10]. Adjustment disorder and stressful events that may result in an AjD diagnosis have been reported to be associated with suicide attempts among adolescents and young adults [12,13]. Therefore, addressing adolescents’ AjD in diverse cultures is an important issue in understanding and supporting youth mental health.

AjD is classified as a stress-related disorder, and the risk for AjD has been found to be associated with various sociodemographic factors and types of stressors. In the Lithuanian adult population sample, female gender, greater age, having a university degree, job-related stressors, and health-related stressors were significantly associated with adjustment disorder [10]. Furthermore, it has been suggested that, in adolescents, rates of mental disorders are determined by factors such as sociodemography, lifestyle characteristics, or the quality of relationships [14]. Examining how stressors experienced by adolescents differ across cultures, and which are the risk factors associated with AjD in adolescents, will provide meaningful insights into the mental health of adolescents. 

Besides sociodemographic factors and stressors, intrapersonal and interpersonal factors are also expected to contribute significantly to the development of AjD. To examine the factors associated with AjD, Lorenz et al. [15] used the socio-interpersonal framework model by Maercker and Horn [16], which was developed for stress–response syndromes. According to the socio-interpersonal framework model, individuals are nested in different levels of social contexts that influence their recovery after extreme stressful events. The first level is the individual level, which includes intrapersonal features or impairments and social affective processes, such as shame, anger, guilt, and loneliness. The second level captures the interaction processes in close relationships, such as social support, empathy, and communication factors. The third level includes societal and cultural factors [16]. Lorenz et al. showed that both intrapersonal and interpersonal factors contributed to the diagnosis of AjD in a sample of laid-off adults (*n* = 316) in Zurich [15]. The current study examines the association between intra-interpersonal factors and adolescent AjD in terms of the social-interpersonal framework model. 

Regarding the intrapersonal factor, we focus on individual resilience. Lee et al. [17] suggest that resilience counteracts the effects of trauma and adversity on mental and physical health. In addition, a meta-analytic study has demonstrated that adolescents’ trait resilience is associated with their mental health [18]. For interpersonal factors, it is suggested that loneliness, as a social affective reaction (first level), and perceived social support, as an interaction process in close relationships (second level), are each associated with an AjD diagnosis [15]. 

Stemming from the lack of ICD-11 adjustment disorder studies in adolescence in general, and from a cross-cultural perspective, we aimed to (1) examine the prevalence of AjD in Japanese and Lithuanian adolescents; and (2) identify the factors associated with AjD in terms of the socio-interpersonal framework model. Specifically, we examined the effects of age, gender, socioeconomic status, and cumulative stressors as societal and cultural factors, resilience as an intrapersonal factor, and loneliness and perceived support as interpersonal factors on adolescent AjD. For the societal and cultural factors, we expected that female gender, greater age, greater socioeconomic difficulties, and greater cumulative stressors contributed to AjD. In addition, we hypothesized that higher loneliness, lower perceived support, and resilience were negatively associated with the AjD. 

## 2. Materials and Methods

### 2.1. Participants and Procedures

This study is part of the larger multicultural longitudinal study Stress and Resilience in Adolescence (STAR-A) initiated at the Center for Psychotraumatology at Vilnius University in Lithuania. This study is also part of a project at Hiroshima University in Japan that examines the relationship between youth trauma experiences, resilience, and identity development. Ethical approval was obtained from the relevant Institutional Review Boards in both Lithuania and Japan. Consent from participants and informed parental or official guardian consent was obtained prior to data collection. In this paper, data from the third wave of STAR-A conducted in Lithuania and the first wave of STAR-A-JP conducted in Japan were analyzed due to the similar timing of the two studies. This study was based on a cross-sectional assessment.

Data collection in Lithuania took place in various regions from March to June 2021. Due to COVID-19 pandemic restrictions and lockdown, data were collected using a platform for online surveys. In collaboration with each school, data collection times were set up. The researcher or trained student researcher explained the procedures to the adolescent groups in online meetings and answered the questions while participants were filling out surveys. We approached 1299 Lithuanian adolescents from 49 schools to participate in the study, and 854 adolescents filled in an online survey with a response rate of 65.7%. 

In Japan, data collection took place between June and July 2021. Adolescents from various regions of Japan completed an online survey through Lancers (https://www.lancers.jp/?ref=co, accessed on 22 June 2021), one of Japan’s leading crowdsourcing sites with the largest number of registered users. As adolescents under the age of 18 cannot register themselves with Lancers, registrants who have a child(ren) in middle school or high school received notification of a call for survey participation, including the procedure of this study and a hyperlink to the online survey. Registrants’ children then answered the survey and received 1000 JPY (approximately 8.80 USD) for their participation. For the Japanese sample, the response rate was not available as the recruitment was via a large panel of participants of the survey company.

After excluding 27 participants because of missing data and careless responding, the final study sample consisted of 1745 adolescents from Japan (*n* = 913) and Lithuania (*n* = 832), 49.8% being female, and the mean age (SD) being 15.52 (1.64); the age range was 12–18. The participants’ characteristics in each country are presented in Table 1. 

We conducted one-year studying period of the prevalence of AjD. Considering the ICD-11 diagnostic criteria for AjD [1], its symptoms may persist for six months, and if the stressor persists, the symptoms may be even more prolonged. Therefore, we asked participants whether they had experienced stressors in the past year.

### 2.2. Measures

#### 2.2.1. Adjustment Disorder Symptoms

Adjustment disorder symptoms were assessed using the Children and Adolescent version of the brief Adjustment Disorder New Module-8 (ADNM-8) scale [19]. The ADNM-8 is a brief version of the ADNM-20 [3] and measures the core AjD symptoms of preoccupation and failure to adapt. The ADNM-8 used in the study comprises two parts: a list of 17 life stressors relevant to children and adolescents (e.g., parental divorce, school change, change of living place, etc.), and 8 items measuring the core AjD symptoms. In the first part, participants were asked to indicate which from the list of 17 stressors they recognized as a significant stressor in the past year. The list of stressors was updated and adjusted for the adolescent sample by the authors of the study. In the second part, participants were asked to indicate how often the respective symptom items applied to them on a 4-point Likert scale: 1 = never; 2 = rarely; 3 = sometimes; 4 = often. The four ADNM-8 symptom items measured preoccupation with the stressor and the thoughts revolving around the stressor. The remaining four ADNM-8 symptom items measured a failure to adapt, including difficulties concentrating, sleep disturbances, withdrawal from close ones, and difficulties in carrying out daily activities or work. The same version of ADNM-8 was used as in previous studies with adult samples.

Since the ADNM-8 was used in a Japanese sample for the first time, the ADNM-8 was translated into Japanese using the back-translation procedure; it previously was used in a Lithuanian sample [10,19]. The internal reliability of the total scores in the total sample was good (Japanese version = 0.93; Lithuanian version = 0.93), as were the internal reliability estimates for the preoccupation (Japanese version = 0.90; Lithuanian version = 0.91) and failure to adapt (Japanese version = 0.85; Lithuanian version = 0.84) subscale scores. 

Several studies used a cut-off score of ≥23 for the total ADNM-8 symptoms to identify a probable adjustment disorder diagnosis based on previous studies in Lithuania [20,21,22]. Due to the lack of ADNM-8 studies in adolescence, we used a data-driven approach to identify groups of adolescents with high symptoms of AjD based on the ADNM-8 scores using a latent class analysis approach.

#### 2.2.2. Societal and Cultural Factors

Participants were asked to answer eight questions about sociodemographic factors. Specifically, participants responded to the following questions: nationality, age, gender (0 = girl; 1 = boy; 2 = other), adults they live with (both parents; mother only; father only; other relatives; foster parent; live in an institution), parents’ employment status (“Is your mother/father currently working?”: 0 = “yes”; 1 = “no”; 2 = “I don’t know”), socio-economic difficulties (“I find that my family can mostly afford to buy what we need”: 0 = “absolutely true”: 1 = “quite true”; 2 = “not quite true”; 3 = “absolutely false”), and received psychological help in the past year (“Have you received psychological help in the last year?”: 0 = “No”; 1 = “Yes, I visited a counsellor one or more times”; 2 = “Yes, I went to a counsellor for a few months or more”) (see Table 1 for details). 

#### 2.2.3. Resilience

Resilience was measured by the Resilience Scale RS-14 [23]. Participants were asked to indicate how they relate to each statement on a 7-point Likert scale: 1 = totally disagree; 7 = totally agree. Resilience was calculated by summing the responses to all the RS-14 items’ scores. Higher scores indicate a higher level of resilience. In this study, we used the Lithuanian RS-14 version [24] for a Lithuanian sample and the Japanese RS-14 version [25] for a Japanese sample. The internal reliability of the total scores in a total sample was good (Japanese version = 0.90; Lithuanian version = 0.91).

#### 2.2.4. Loneliness

Loneliness was measured by a three-item scale [26]. Participants were asked how often they feel like (1) they are missing being with other people; (2) left behind others; and (3) isolated from others. Possible answers were “Never” (=0); “Sometimes” (=1); and “Often” (=2). Loneliness was calculated by summing all three items’ scores. Higher scores indicate a higher level of loneliness. The internal reliability of the loneliness scale was good (α = 0.99; Lithuanian version, α = 0.99; Japanese version, α = 0.80).

#### 2.2.5. Perceived Positive Social Support

Perceived positive social support (PPSS) was measured by using a revised version of the Crisis Support Scale (CSS) [27]. We selected four items to measure PPSS: (1) how often does someone tend to listen if the participant wants to talk; (2) can the participant talk about his thoughts and feelings with others; (3) do people sympathize and support the participant; and (4) does anyone help the participant with everyday practical problems. Each item was evaluated using a 7-point Likert scale, from “Never” (=1) to “Always” (=7). PPSS was calculated by totalizing all four items’ scores. Higher scores indicate a higher PPSS. The internal reliability of the PPSS scale was good (α = 0.99; Lithuanian version, α = 0.99; Japanese version, α = 0.88).

### 2.3. Data Analysis

As preliminary analyses, we conducted the confirmatory factor analyses (CFA) and measurement invariance analyses of the ADNM-8. The results confirmed sufficient factorial validity and scalar invariance between the two countries. The detailed information on the validity of the ADNM-8 is reported in the Supplementary Material (Sections S1 and S2). We conducted CFA and measurement invariance analyses using Mplus 8.7 (Muthén & Muthén, Los Angeles, CA, USA), multinomial logistic regression, and other data analyses using SPSS Statistics 27 (IBM Inc., Armonk, NY, USA). 

First, we conducted comparative analyses between the life stressors among Japanese and Lithuanian adolescents. The proportions of adolescents who experienced each stressor, the proportion of adolescents who had experienced one or more stressor(s), and the total number of stressors experienced were compared between Japan and Lithuania.

To identify the high risk for the probable ICD-11 adjustment disorder in the adolescent sample, we applied the Latent Class Analysis (LCA) approach [28]. For the sum scores of the ADNM-8, two indicators of adjustment disorder symptoms—preoccupation and failure to adapt—were included in the LCA. We conducted the LCA in Lithuania and Japan separately. To decide on the number of latent classes, we used the Akaike Information Criterion (AIC) and Bayesian Information Criterion (BIC) statistics, where a solution with k classes should be lower than a solution with k-1 classes; a statistically significant *p*-value of the adjusted Lo–Mandel–Rubin test, and the Entropy score, with values equal or above 0.70, were indicative of an accurate classification. After identification of high-risk adjustment disorder groups in both samples using LCA, the means of the ADNM-8 symptoms scores in the identified LCA classes were used as the cut-off scores of the ADNM-8 for probable adjustment disorder in the Lithuanian and Japanese samples.

Multinomial logistic regression was used to examine the risk factors associated with AjD in adolescents who reported experience of at least one significant life stressor. Nationality, gender, age, socioeconomic difficulties, cumulative stressors, loneliness, positive social support, and resilience were entered into the model as risk factors. We conducted multinomial logistic regression twice for the purpose of changing the reference group. The first reference group was the no-risk group, and the second was the risk group. Cox and Snell and Nagelkerke determination pseudo-coefficient *R*^2^ values were used to explain the general percentage of data variance of the multinomial logistic regression.

## 3. Results

### 3.1. Stressors in Japan and Lithuania

Most adolescents in the total sample (73.5%) reported having experienced at least one stressor in the past year (71.1% in Japan; 76.2% in Lithuania). The mean value of the number of stressors experienced was 2.17 (SD = 2.23) in the total sample. The number of cumulative stressors experienced in the Lithuanian sample (M = 2.38, SD = 2.27) was higher than that of the Japanese sample (M = 1.97, SD = 2.18) (*t*(*df*) = 3.84 (1712.80), *p* < 0.001). As shown in Table 2, there were also significant differences between Japan and Lithuania in the percentage of adolescents who experienced each stressor. Japanese have experienced more financial problems in family, serious family conflicts, and other stressful events compared to the Lithuanian sample. In turn, Lithuanians have experienced more school change, moving to another country, one or both parents/foster parents moving to live in another country, death of a close family member, illness of a close family member, the birth of a sibling, end of a friendship, end of a relationship with boyfriend/girlfriend, difficulties in school, and suicide attempt of a loved one.

### 3.2. The Prevalence of AjD

The LCA analyses indicated that the four classes solution fitted the data best in both countries (see Table 3). The least numerous classes with the highest scores of the preoccupation and failure to adapt were labeled as ‘Clinical’. The cut-off score of the ADNM-8 symptom scale in both countries was found to be ≥28 for the Clinical group class. The second class, with still relatively high scores on the ADNM-8 subscales, was labeled as ‘Probable’ AjD, with cut-off scores of ADNM-8 ≥ 22 and ADNM-8 ≥ 21 for the Lithuanian and Japanese samples, respectively. We used this empirically driven ADNM-8 cut-off score for risk of adjustment disorder in our further analysis. The remaining two classes were labeled as ‘Low-symptom’ and ‘No-symptom’.

Using the cut-off scores by the LCA analysis, in a total sample of adolescents who experienced at least one life stressor (*n* = 1283), 286 participants (22.3%) were found to be in the Probable AjD group; 21.6% of Lithuanians exceeded the ADNM-8 score of ≥22, and 23.0% of the Japanese participants exceeded the ADNM-8 score of ≥21. There was no significant difference between Lithuanian and Japanese adolescents in the prevalence of AjD risk (*χ*^2^ (*df*) = 1.71 (1), *p* = 0.192). However, for the clinical cut-off (the ADNM-8 scores ≥ 28), a significant difference between Lithuanian (11.7%) and Japanese (6.9%) adolescents (*χ*^2^ (*df*) = 8.56 (1), *p* = 0.014) was found. 

### 3.3. The Risk Factors Associated with AjD

Multinomial logistic regression was conducted to examine which factors (nationality, gender, age, socioeconomic difficulties, cumulative stressors, resilience, loneliness, and perceived positive social support) were associated with an AjD diagnosis (Table 4). Model Likelihood Ratio Tests showed a good model fit (*χ*^2^ (*df*) = 366.27 (16), *p* <0.001). Cox and Snells’ and Nagelkerke’s determination pseudo coefficient *R*^2^ values were, respectively, 0.248 and 0.309. 

Table 4 reports the adjusted odds ratios (OR) from the multinomial logistic regression. Statistically significant risk factors of the probable AjD group compared to the no risk group were female gender, greater socioeconomic difficulties, cumulative stressors, loneliness, lower perceived positive social support, and lower resilience. As risk factors of the AjD clinical group compared to the no-risk group, revealed a Lithuanian nationality, female gender, cumulative stressors, loneliness, lower perceived positive social support, and lower resilience. Furthermore, statistically significant risk factors of the AjD clinical group compared to the probable AjD risk group were Lithuanian nationality, cumulative stressors, and lower resilience. 

## 4. Discussion

The current study aimed to (1) examine the prevalence of AjD in Japanese and Lithuanian adolescents; and (2) identify the risk factors associated with adolescent AjD in terms of the socio-interpersonal framework model. Results showed a high prevalence of AjD risk in adolescents, and further showed that societal and cultural factors, intrapersonal factors, and interpersonal factors were associated with AjD risk. Therefore, the hypotheses were generally supported.

### 4.1. The Prevalence of AjD and Stressors in Japanese and Lithuanian Adolescents

A high proportion of adolescents exceeded the cut-off value for probable AjD (22.3%). This high prevalence of AjD in this study could be influenced by the COVID-19 pandemic. Recent studies in both Japan and Lithuania showed that the COVID-19 pandemic and resulting social restrictions were associated with behavioral and emotional problems in adolescents [29,30]. There is also increasing evidence at the global level that the COVID-19 pandemic has a negative impact on the mental health of adolescents [31,32]. 

The results of this study indicated that there was no significant difference between Lithuanian and Japanese adolescents in the prevalence of probable AjD. However, in terms of the cut-off values for more severe symptoms, the prevalence of clinical AjD was significantly higher in Lithuanian adolescents than in Japanese adolescents. Given the finding of a previous study [10], this difference could be influenced by the higher number of cumulative stressors of Lithuanians compared to Japanese adolescents. Additionally, Lithuanian adolescents reported higher exposure to some specific stressors, such as parental emigration. Previous studies showed that adolescents with parents abroad reported higher levels of various mental health issues in comparison to other adolescents [33]. Higher exposure to interpersonal stressors, such as the death or illness of a close family member, as well as the end of a friendship or a relationship with a boyfriend/girlfriend, also was reported in the Lithuanian sample compared to the Japanese sample. Previous studies show that interpersonal relationship problems may contribute to higher loneliness in adolescence [34], which, in turn, may foster mental health issues [35], as was found in the current study. 

On the other hand, Japanese adolescents have experienced more financial problems in family, serious family conflicts, and other stressful events as compared to the Lithuanian adolescents. Since Japan implemented a “mild lockdown” [36], relying on voluntary public cooperation during the pandemic, it can be assumed that the social restrictions on people were less severe than in Lithuania. Despite this, it has been suggested that lifestyle changes in Japan, such as self-restraint from going out and increased teleworking, have led to an increase in marital domestic violence [37,38], a record number of child abuse cases [39], and a general decline in consumer activity [40]. Lifestyle changes during the COVID-19 pandemic may have led to serious family-related stressors for Japanese adolescents. 

### 4.2. Risk Factors of AjD 

Female gender, greater socioeconomic difficulties, greater cumulative stressors, lower resilience, higher loneliness, and lower positive social support contributed to being at risk of probable AjD (vs. no risk) in adolescents. The results that the female gender was associated with AjD symptoms are in line with previous findings from adult samples [10,19,41]. Meta-analytic studies also have demonstrated that adolescents’ subjective SES and trait resilience are associated with their mental health [18,42]. Furthermore, consistent with previous findings in adults [15,41], perceived social support and loneliness were significantly associated with probable AjD risk. The results of this study suggest that sociocultural, intrapersonal, and interpersonal factors are all associated with AjD symptoms, as they are in adults. For the clinical group (vs. probable AjD), only Lithuanian nationality, greater cumulative stressors, and lower resilience remained significant risk factors, indicating that for more severe symptoms, sociocultural and intrapersonal factors may be more influential for adolescent AjD.

### 4.3. Limitations and Future Directions 

Although promising results were obtained, the current study has several limitations. First, the cross-sectional design chosen for this study could not address changes in adjustment disorder symptoms and potential risk factors of AjD. Longitudinal studies are needed to assess further the association between adjustment disorders and potential risk factors. Second, it is possible that other factors not addressed in this study may contribute to the development of AjD. For example, Lorenz et al. [15] showed that in addition to loneliness and social support, higher dysfunctional disclosure and lower self-efficacy were associated with both higher symptom severity and higher likelihood of meeting the diagnostic criteria for AjD. Including these variables, risk factors associated with the development of AjD in adolescents need to be examined in more detail. Third, this study was conducted as an online survey due to the spread of COVID-19. Since this study included adolescents from diverse geographic regions and social classes, and there are previous studies that support the reliability of online surveys [43], there is some generalizability of our results. However, there are some problems with the Japanese data, such as the possibility of bias in the regions of the respondents and the lack of control over the environment in which participants responded to the survey. Therefore, future studies should examine the prevalence of AjD among adolescents and their risk factors by conducting face-to-face surveys. Finally, this study dealt only with the number of stressors as a risk factor for AjD and not with the impact of stressors. Considering the findings that the impact of stressors varies by culture [44], it will be necessary to conduct cross-cultural comparisons of the impact of stressors and their impact on AjD in the future.

## 5. Conclusions

The current study provided scientific evidence on the prevalence and risk factors associated with ICD-11 AjD in adolescents. Specifically, the prevalence of AjD among adolescents was relatively high in both Japan and Lithuania. In addition, sociocultural (female gender, socioeconomic difficulties, and cumulative stressors), intrapersonal (lower resilience), and interpersonal factors (loneliness, lower perceived positive social support) were risk factors of probable AjD risk (vs. no risk) in adolescents from both countries. To understand the patterns of onset of specific mental disorders and the context in which they occur, it is important to examine which factors affecting mental disorders are characteristic of each culture and which factors are common across cultures. Future comparative studies among multiple cultures will contribute to the existing knowledge about the onset, patterns, and consequences of AjD.

## Figures and Tables

**Table 1:** Demographic characteristics of the participants (*n* = 1745).

Demographic Characteristics	Total (*n* = 1745)		Japanese (*n* = 913)		Lithuanians (*n* = 832)		*χ*^2^(1)
	*n*	%	*n*	%	*n*	%	
Age: M (SD)	15.52 (1.64)		14.93 (1.67)		16.17 (1.34)		
Range	12–18		12–18		13–18		
Gender							
Female	869	49.8%	376	41.2%	493	59.3%	56.87 ***
Male	876	50.2%	537	58.8%	339	40.7%	
Adult lives with							
both parents or foster parents	1421	81.4%	825	90.4%	596	71.6%	103.57 ***
One parent	298	17.1%	77	8.4%	221	26.6%	
Other (e.g., relatives, institution)	26	1.5%	11	1.2%	15	1.8%	
Mother working							
Yes	1387	79.5%	656	71.9%	731	87.9%	231.50 ***
No	106	6.1%	21	2.3%	85	10.2%	
Do not know	252	14.4%	236	25.8%	16	1.9%	
Father working							
Yes	1582	90.7%	868	95.1%	714	85.8%	50.68 ***
No	65	3.7%	26	2.8%	39	4.7%	
Do not know	98	5.6%	19	2.1%	79	9.5%	
Family can mostly afford to buy what they need							
Absolutely true	738	42.3%	174	19.1%	564	67.8%	447.80 ***
Quite true	858	49.2%	602	65.9%	256	30.8%	
Not quite true	132	7.6%	122	13.4%	10	1.2%	
Absolutely false	17	1.0%	15	1.6%	2	0.2%	
Received psychological help in last year							
No	1604	91.9%	875	95.8%	729	87.6%	42.71 ***
Yes, visited counselor one or more times	97	5.6%	31	3.4%	66	7.9%	
Yes, went to a counselor for a few months or more	44	2.5%	7	0.8%	37	4.4%	

Note: M = mean; SD = standard deviation. *** *p* < 0.001.

**Table 2:** Life stressors among Japanese and Lithuanian adolescents (*n* = 1745).

Stressors	Total (*n* = 1745)		Japanese (*n* = 913)		Lithuanians (*n* = 832)		*χ*^2^(1)	*p*
	*n*	%	*n*	%	*n*	%		
ADNM-8 stressors								
Parental divorce	64	3.7%	30	3.3%	34	4.1%	0.79	0.374
School change	133	7.6%	34	3.7%	99	11.9%	41.32	<0.001
Change of living place/moving to a new home	120	6.9%	61	6.7%	59	7.1%	0.11	0.735
Moving to another country	17	1.0%	13	1.4%	4	0.5%	4.01	0.045
One or both parents/foster parents moving to live in another country	41	2.3%	10	1.1%	31	3.7%	13.13	<0.001
Death of a close family member	277	15.9%	119	13.0%	158	19.0%	11.57	<0.001
Illness of a close family member	343	19.7%	162	17.7%	181	21.8%	4.44	0.035
Own serious illness	77	4.4%	40	4.4%	37	4.4%	0.00	0.947
Bullying	147	8.4%	69	7.6%	78	9.4%	1.86	0.172
Birth of a sibling	53	3.0%	18	2.0%	35	4.2%	7.39	0.007
Financial problems in family	229	13.1%	148	16.2%	81	9.7%	16.01	<0.001
End of a friendship	499	28.6%	174	19.1%	325	39.1%	85.32	<0.001
End of relationship with boyfriend/girlfriend	195	11.2%	73	8.0%	122	14.7%	19.50	<0.001
Serious family conflicts	407	23.3%	240	26.3%	167	20.1%	9.40	0.002
Difficulties in school	681	39.0%	278	30.4%	403	48.4%	59.2	<0.001
Suicide attempt of a loved one	76	4.4%	12	1.3%	64	7.7%	42.51	<0.001
Other stressful events	426	24.4%	321	35.2%	105	12.6%	119.84	<0.001
At least one stressor	1283	73.5%	649	71.1%	634	76.2%	5.86	0.016
Cumulative stressors								
1 stressor	380	21.8%	217	23.8%	163	19.6%	4.46	0.035
2–3 stressors	523	30.0%	260	28.5%	263	31.6%	2.04	0.154
4–5 stressors	236	13.5%	108	11.8%	128	15.4%	4.71	0.030
6 or more stressors	144	8.3%	64	7.0%	80	9.6%	3.90	0.048
Mean (SD)	2.17 (2.23)		1.97 (2.18)		2.38 (2.27)		*t*(*df*) = 3.84 (1712.80)	<0.001

**Table 3:** Model fit indices of the latent class analyses.

Model	Loglikelihood	AIC	BIC	Entropy	BLRT *p*-Value	LMR-A *p*-Value
2 classes	−4335.257	8684.515	8718.232	0.762	<0.001	<0.001
3 classes	−4142.600	8305.201	8353.368	0.831	<0.001	<0.001
4 classes	−4073.394	8172.789	8235.407	0.843	<0.001	0.003
5 classes	−4049.002	8130.004	8207.072	0.847	<0.001	0.058

Note: AIC = Akaike Information Criterion; BIC = Bayesian Information Criterion; BLRT = Bootstrap Likelihood Ratio Test; LMR-A = Lo–Mendell–Rubin Adjusted Likelihood Ratio Test.

**Table 4:** Multinomial logistic regression for AjD prediction (*n* = 1283).

Variable	Probable AjD (*n* = 286) vs. No Risk (*n* = 878)			Clinical (*n* = 119) vs. No Risk			Clinical vs. Probable AjD		
	B (SE)	*p*	OR (95% CI)	B (SE)	*p*	OR (95% CI)	B (SE)	*p*	OR (95% CI)
Nationality (Lithuanian)	0.20 (0.20)	0.318	1.22 (0.83–1.80)	1.12 (0.31)	<0.001	3.05 (1.65–5.63)	0.92 (0.32)	0.004	2.50 (1.34–4.67)
Gender (Girl)	0.61 (0.15)	<0.001	1.85 (1.37–2.50)	1.10 (0.26)	<0.001	3.01 (1.82–4.98)	0.49 (0.26)	0.063	1.63 (0.97–2.73)
Age	0.05 (0.05)	0.838	1.05 (0.95–1.15)	0.06 (0.08)	0.428	1.07 (0.91–1.24)	0.02 (0.08)	0.826	1.02 (0.87–1.19)
SES (Difficulties)	0.28 (0.12)	0.026	1.32 (1.03–1.68)	0.30 (0.19)	0.117	1.35 (0.93–1.97)	0.03 (0.19)	0.900	1.03 (0.70–1.50)
Cumulative Stressors	0.21 (0.04)	<0.001	1.24 (1.15–1.33)	0.43 (0.50)	<0.001	1.54 (1.40–1.69)	0.22 (0.05)	<0.001	1.24 (1.13–1.36)
Resilience	−0.03 (0.01)	<0.001	0.97 (0.96–0.99)	−0.08 (0.01)	<0.001	0.92 (0.90–0.94)	−0.06 (0.01)	<0.001	0.95 (0.93–0.96)
Loneliness	0.06 (0.02)	<0.001	1.07 (1.03–1.10)	0.09 (0.02)	<0.001	1.09 (1.05–1.14)	0.03 (0.02)	0.230	1.03 (0.98–1.07)
PPSS	−0.06 (0.01)	<0.001	0.94 (0.91–0.96)	−0.09 (0.02)	<0.001	0.92 (0.88–0.96)	−0.03 (0.02)	0.238	0.98 (0.94–1.02)

Note: B = unstandardized regression coefficient; OR = odds ratio; CI = confidence interval; SES = socio-economic status; PPSS = perceived positive social support. Model fit: Model Likelihood Ratio Tests (*χ*^2^ (*df*) = 366.27 (16), *p* < 0.001) showed a good model fit. Nagelkerke’s determination pseudo coefficient *R*^2^ = 0.31.

## Data Availability

Not applicable.

## Supplementary Materials

The following supporting information can be downloaded at: https://www.mdpi.com/article/10.3390/brainsci12091172/s1, Section S1: Preliminary analyses: Validation and Measurement Invariance of ADNM-8; Section S2: CFA and Measurement Invariance Results for ADNM-8.
