# Supplementary material for: Cross-Cultural Comparison of ICD-11 Adjustment Disorder Prevalence and Its Risk Factors in Japanese and Lithuanian Adolescents
Source: Brain Sci. 2022 Aug 31;12(9):1172. doi: 10.3390/brainsci12091172 (PMC9496776; doi:10.3390/brainsci12091172)
Supplement: Supplementary file 1 [file brainsci-12-01172-s001.zip › brainsci-1833857-supplementary.pdf]

Supplementary Material

Section S1: Preliminary analyses: Validation and Measurement Invariance of ADNM-8

Using CFA, we examined a two-factor model for the ADNM-8 consisting of the preoccupation and failure to adapt in the subsample of adolescents that experienced at least one life stressor. We conducted the CFA in Lithuanian and Japanese subsamples separately as well as in a total study sample. The CFA models were estimated using the Maximum Likelihood Robust (MLR) estimator, which can address variables with slight deviations from a normal distribution. These models' fits were assessed using the Chi-square test, the root-mean-square error of approximation (RMSEA), the comparative fit index (CFI), Tucker Lewis index (TLI), and the standardized root mean square residual (SRMR) indices. RMSEA and SRMR values of ≤0.08, CFI and TLI values ≥0.90, and a non-significant chi-square result indicate a good model fit.

To check whether the ADNM-8 assesses the same construct across Japanese and Lithuanian samples, we tested its measurement invariance across the two countries. We tested three levels of measurement invariance [1]: configural (factor structures are equivalent across countries), metric (factor structures and loadings are equivalent across countries), and scalar invariance (factor structures, loadings and item intercepts are equivalent across countries). Model comparisons were conducted by examining the changes in fit indices. ΔCFI ≤ -0.010 and ΔRMSEA ≤ 0.015 indicate no significant differences between models.

Section S2: CFA and Measurement Invariance Results for ADNM-8

The initial correlated two factor model did not have a good fit in a total sample: χ^2 (*df*) = 218.23 (19), $p < 0.001$, RMSEA = 0.090 (90% CI 0.080–0.101), SRMR = 0.029, CFI = 0.956, TLI = 0.935 (χ^2(*df*) = 126.06 (19), $p < 0.001$, RMSEA = 0.093 (90% CI 0.078–0.109), SRMR = 0.035, CFI = 0.948, TLI =

0.924 in Japan, $\chi^2(df) = 137.15$ (19), $p < 0.001$, RMSEA = 0.099 (90% CI 0.084–0.115), SRMR = 0.028, CFI = 0.954, TLI = 0.931 in Lithuania). Considering modification indices, the model was revised by adding the correlation between the 2 ADNM-8 item errors (items 1 and 2) of the preoccupation latent factor. The revised model fitted the data well: χ^2 (df) = 109.63 (18), $p < 0.001$, RMSEA = 0.063 (90% CI 0.052–0.075), SRMR = 0.024, CFI = 0.980, TLI = 0.968 (χ^2 (df) = 84.82 (18), $p < 0.001$, RMSEA = 0.076 (90% CI 0.060–0.092), SRMR = 0.029, CFI = 0.968, TLI = 0.950 in Japan, χ^2 (df) = 52.78 (18), $p <$ 0.001, RMSEA = 0.055 (90% CI 0.038–0.073), SRMR = 0.025, CFI = 0.986, TLI = 0.979 in Lithuania. This revised model was also confirmed in a previous study [19].

As the result of the measurement invariance test of the ADNM-8, the configural, met-ric, and scalar invariant models of the ADNM-8 demonstrated a good fit. The changes between configural and metric models were ΔCFI = 0.004, and ΔRMSEA = 0.001. The changes between metric and scalar models were ΔCFI = 0.008, and ΔRMSEA = 0.005. Thus, scalar invariance was confirmed between two countries.
